# Point-of-care ultrasound in obstetrics and gynecology: latest advancements and evolution

**DOI:** 10.1007/s00404-026-08584-z

**Published:** 2026-10-14

**Authors:** Florian Recker

**Affiliations:** 1 Department of Obstetrics and Prenatal Medicine, University Hospital Bonn, Venusberg Campus 1, 53127 Bonn, Germany; 2 Department of Obstetrics, Faculty of Medicine and, University of Cologne, University Hospital Cologne, Kerpener Street 34, 50931 Cologne, Germany

**Keywords:** Point-of-care ultrasound, Obstetrics, Gynecology, Handheld ultrasound, Artificial intelligence, Tele-ultrasound, Medical education

## Abstract

Point-of-care ultrasound (POCUS) in obstetrics and gynecology has evolved from a pragmatic bedside adjunct into a rapidly expanding clinical, educational and technological field. Since the publication of earlier narrative reviews, the discipline has been reshaped by handheld ultrasound systems, wireless probes, cloud-based image management, tele-ultrasound, artificial intelligence (AI) for image acquisition and interpretation, and the first specific practice guidance for POCUS in obstetrics and gynecology. This narrative review summarizes recent advances and proposes a clinically useful framework for the European ultrasound community. POCUS should be understood as a focused, problem-oriented and immediately actionable ultrasound examination performed and interpreted by the treating clinician. It is not a replacement for guideline-based screening, detailed fetal anatomy assessment, comprehensive transvaginal gynecologic imaging, Doppler surveillance or expert ultrasound. The strongest evidence supports POCUS for early pregnancy triage, confirmation of fetal viability and presentation, basic assessment of placental location and amniotic fluid, intrapartum evaluation of fetal head position and selected postpartum or gynecologic emergencies. In gynecology, POCUS can accelerate triage of acute pelvic pain, suspected ectopic pregnancy, intrauterine device position and adnexal pathology, but definitive risk stratification of complex masses remains a task for standardized specialist sonography. Technological progress has been substantial: handheld devices now provide diagnostically useful images for several focused tasks, AI-enabled blind-sweep protocols can estimate gestational age and fetal malpresentation in low-resource settings, and tele-ultrasound can extend expert support beyond tertiary centers. The next phase of OB/GYN POCUS will depend less on device availability than on implementation: indication-specific protocols, formal training, competency assessment, documentation, image archiving, infection control, data protection, quality assurance and clear escalation rules. POCUS in obstetrics and gynecology should therefore be framed as a governed clinical pathway rather than a device-driven shortcut.

## Key points

- POCUS in OB/GYN is a focused clinical examination for immediate decision-making, not a substitute for comprehensive diagnostic ultrasound.
- The strongest current evidence supports early pregnancy triage, fetal viability and presentation, selected intrapartum questions, and pragmatic gynecologic triage.
- Handheld devices, AI-enabled blind sweeps and tele-ultrasound have expanded the practical reach of POCUS, especially outside tertiary ultrasound units.
- Implementation requires a governance model: indications, training, competency assessment, documentation, image archiving, infection control, data protection and escalation pathways.
- The curriculum, credentialing model and governance framework proposed in this review are an expert-informed proposal intended for discussion and local adaptation, not a formally validated or consensus-based European standard.

## Introduction

Ultrasound is central to modern obstetrics and gynecology, yet access to high-quality sonography remains uneven across clinical settings. In tertiary perinatal centers, ultrasound is frequently performed by highly trained specialists with advanced equipment, structured documentation and embedded quality assurance. In contrast, emergency departments, labor wards, peripheral clinics, rural settings and low-resource regions often require rapid, clinically focused answers before a formal ultrasound appointment is available. This clinical gap is the conceptual space in which point-of-care ultrasound (POCUS) has become increasingly important.

The 2021 review by Recker and colleagues described POCUS in obstetrics and gynecology as a focused bedside extension of the physical examination that can support time-critical decisions while explicitly not replacing detailed prenatal or diagnostic ultrasound [1]. That framing remains valid. However, the field has changed considerably. Handheld systems have become cheaper, more mobile and easier to integrate into clinical workflows. Wireless probes, cloud-based archiving, DICOM interfaces and telemedicine platforms have reduced the technical distance between bedside imaging and formal image management. At the same time, artificial intelligence (AI) has moved from retrospective image analysis to prospectively evaluated and in some settings regulatory-cleared workflows for automated gestational age estimation and simplified acquisition.

The clinical question has therefore shifted. Five years ago, the key issue was whether portable ultrasound machines could generate images good enough to support focused OB/GYN decisions. Today, the more important issue is whether POCUS can be implemented safely, reproducibly and equitably within a governed clinical pathway. This question is especially relevant for the European ultrasound community, which has long emphasized training, staged competence, quality standards, safety and professional stewardship. This narrative review updates the clinical, technological and educational evidence and proposes an implementation-oriented framework for OB/GYN POCUS.

### Literature identification and selection

This article is a narrative, non-systematic review. Literature was identified through structured, but deliberately non-exhaustive searches of PubMed/MEDLINE, Embase, the Cochrane Library and Scopus, complemented by targeted screening of the websites of professional societies and standards bodies (ISUOG, EFSUMB, DEGUM, WHO, RCOG, ACOG, AWMF and BMUS), by hand searching of the reference lists of retrieved articles, and by regulatory and manufacturer announcements for device- and AI-related developments that are not yet represented in the indexed literature. The principal search period was January 2020 to March 2026, chosen so that this review covers developments published after the earlier narrative review of the same topic [1]; a small number of older landmark publications were retained where these define the concept of point-of-care ultrasound, the boundaries of handheld imaging or the methodological basis of competency assessment. Search terms combined “point-of-care ultrasound”, “POCUS”, “handheld ultrasound”, “portable ultrasound”, “pocket-sized ultrasound”, “tele-ultrasound”, “artificial intelligence” and “blind sweep” with “obstetrics”, “gynecology”, “pregnancy”, “early pregnancy”, “intrapartum”, “labor”, “adnexal”, “intrauterine device”, “training”, “curriculum”, “competency” and “governance”. Publications in English and German were considered.

Sources were selected thematically rather than according to a predefined methodological hierarchy. Priority was given to four categories: practice guidance, position papers and quality standards issued by ultrasound or obstetric and gynecologic societies; studies validating focused or handheld examinations against standard sonography for questions that a treating clinician might reasonably ask at the bedside; prospectively evaluated or regulatory-cleared artificial intelligence and tele-ultrasound applications relevant to acquisition or interpretation at the point of care; and work on training, competency assessment, credentialing and program governance. Publications concerned with comprehensive specialist sonography, fetal anomaly screening, detailed Doppler surveillance or non-OB/GYN POCUS applications were included only where they help define the boundary of the focused examination. Single case reports, conference abstracts without full-text data and commentaries without new evidence were not considered.

### Definition and conceptual boundaries

POCUS is best defined as the acquisition, interpretation and immediate clinical integration of ultrasound findings by the treating clinician at the time and place of care [2, 3]. It differs from conventional diagnostic ultrasound in scope, timing and purpose. POCUS is limited, focused and problem-oriented; it is usually performed to answer a binary or short-list clinical question, guide an intervention, triage urgency, or monitor change. Comprehensive ultrasound, in contrast, involves systematic assessment, formal documentation, guideline-defined reporting, often a broader differential diagnosis and, in many OB/GYN contexts, specific credentialing.

This distinction is not semantic. In obstetrics, POCUS may be suitable for questions such as "Is there fetal cardiac activity?", "What is the fetal presentation?", "Is the placenta clearly away from the cervix on transabdominal assessment?", "Is there grossly normal or abnormal amniotic fluid?", or "Is the fetal head occiput anterior or posterior during labor?" It is not adequate for a complete fetal anatomy scan, detailed placental accreta spectrum evaluation, fetal growth restriction workup, complex Doppler surveillance, fetal echocardiography or screening for structural anomalies. In gynecology, POCUS may rapidly identify an intrauterine device, obvious free fluid, a large adnexal cyst or a suspicious pelvic mass, but it does not replace expert transvaginal imaging and structured risk stratification of adnexal lesions.

**Fig. 1:**
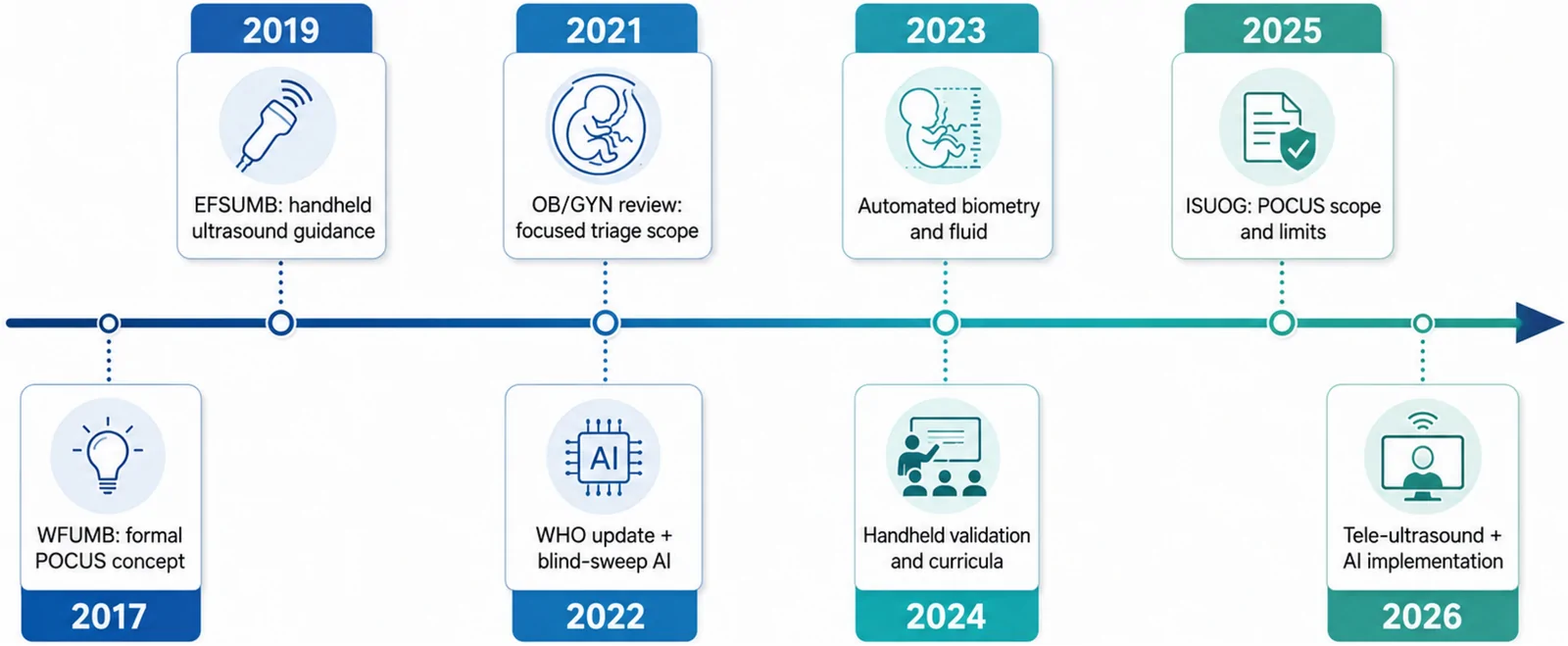
Evolution of point-of-care ultrasound in obstetrics and gynecology from focused bedside access to AI-enabled and governed clinical pathways. The timeline is conceptual and highlights major milestones relevant to recent manuscript framing

European guidance has addressed the same boundary from the device side: the EFSUMB position paper on handheld ultrasound devices frames such systems as an extension of, and not a replacement for, standard equipment, and makes adequate training, documentation and awareness of technical limitations a precondition of their use [4]. Recent practice guidance from ISUOG has made this boundary explicit by framing POCUS as a limited, immediate and indication-dependent examination in obstetric and gynecologic care [5]. Similarly, WHO recommendations support ultrasound in antenatal care for accurate dating and screening before 24 weeks, but this does not mean that any focused bedside scan is equivalent to guideline-based antenatal ultrasound [6]. The European and German quality traditions, including DEGUM standards, reinforce the same principle: POCUS should be integrated into a staged diagnostic system that knows when to stop, document and escalate [7, 8] (Fig. 1).

### Clinical applications in obstetrics

Early pregnancy is the most established OB/GYN POCUS domain. In symptomatic first-trimester patients, delayed differentiation between viable intrauterine pregnancy, pregnancy of unknown location, early pregnancy loss and ectopic pregnancy can lead to prolonged emergency department stays or critical complications. Emergency medicine literature has long shown that focused pelvic ultrasound can improve triage of patients at risk for ectopic pregnancy [9]. More recent syntheses indicate that POCUS in symptomatic first-trimester pregnancy can shorten emergency department length of stay, especially when an intrauterine pregnancy is identified [10]. The clinical value lies in a rapid rule-in strategy: a clearly visualized intrauterine pregnancy with fetal cardiac activity in an appropriate clinical context can substantially change triage. Conversely, a negative or indeterminate transabdominal POCUS examination does not exclude ectopic pregnancy, early pregnancy loss or heterotopic pregnancy. For this reason, uncertain findings require transvaginal ultrasound, serial beta-hCG algorithms and specialist review, consistent with current early pregnancy guidance [11].

Handheld systems have made first-trimester access more convenient, but device limitations are important. Transabdominal handheld ultrasound can be useful from later first trimester onward when a viable intrauterine pregnancy is visible, but early gestations and adnexal pathology often require transvaginal high-frequency imaging. This is one of the major OB/GYN-specific limitations of general POCUS devices: many handheld platforms do not provide a true endocavitary probe, and without transvaginal imaging the diagnostic ceiling in early pregnancy and gynecology is low. A prospective cohort study of handheld transabdominal ultrasound for the confirmation of a vital intrauterine pregnancy in the first trimester illustrates both the feasibility and the ceiling of this approach [12].

In the second and third trimesters, POCUS has strong face validity for focused questions: fetal presentation, fetal number, fetal cardiac activity, placental site, amniotic fluid and gross biometric orientation. Multiple validation studies have found high agreement between portable or handheld devices and conventional systems for selected basic obstetric tasks [13–15]. These findings are clinically relevant for labor wards, triage units, anesthesia teams, remote clinics and low-resource settings. For example, confirming breech presentation before induction, identifying transverse lie before transfer, or documenting fetal cardiac activity after maternal trauma may immediately influence management.

The boundary is again crucial. POCUS can support triage in suspected fetal growth restriction, but it cannot replace formal biometry, growth velocity assessment, Doppler surveillance and etiologic evaluation. Practice bulletins and international initiatives define fetal growth restriction as a longitudinal and pathophysiologic diagnosis, not as a single bedside estimate of fetal size [16, 17]. Similarly, transabdominal POCUS may orient placental location, but suspected placenta previa, vasa previa or placenta accreta spectrum requires transvaginal and/or expert imaging and multidisciplinary planning [18].

Intrapartum ultrasound lies at the intersection of POCUS and specialist obstetric imaging, and the distinction between the two matters more in this domain than in any other discussed here. Comprehensive intrapartum sonography, as described in the ISUOG intrapartum guidelines, is a systematic examination that may include fetal head position and spine orientation, quantitative descent parameters such as angle of progression, head–perineum distance, head–symphysis distance and midline angle, assessment of caput succedaneum and molding, and the use of such parameters within models that estimate the probability of successful operative vaginal delivery [19]. These measurements presuppose dedicated training, standardized planes, reproducible caliper placement and an appreciation of their partly inconsistent predictive performance; they are not established as bedside screening tools. ISUOG guidance emphasizes that intrapartum ultrasound complements, and does not replace, clinical vaginal examination [19]. Recent meta-analytic data suggest potential benefits for selected maternal outcomes, although hard endpoints such as operative delivery rates and neonatal outcomes remain inconsistently improved across studies [20].

Focused intrapartum POCUS is a considerably narrower activity. The assessments that can reasonably be performed as a focused bedside examination by an appropriately trained obstetrician or midwife are those that are binary or categorical, require no measurement and carry immediate consequences: determination of fetal head position as occiput anterior, transverse or posterior before an instrumental delivery is attempted; confirmation of presentation and exclusion of malpresentation; orientation of the second twin after delivery of the first; confirmation of fetal cardiac activity; and a gross impression of amniotic fluid and placental site. Quantitative descent parameters, prediction models for operative vaginal delivery and serial sonographic assessment of labor progression are better regarded as components of comprehensive intrapartum sonography and should be performed by, or under the supervision of, clinicians credentialed for that purpose. Presenting them as POCUS risks transferring an appearance of objectivity to measurements that the bedside operator may not be trained to acquire reproducibly.

The role of focused ultrasound in suspected labor arrest requires particular care. Ultrasound can contribute objective information when digital examination is uncertain, most importantly the position of the fetal head and the confirmation that presentation is as assumed. It cannot by itself establish that labor has arrested, and it cannot identify the cause. Labor arrest is a clinical diagnosis defined by cervical dilatation and descent over time in relation to uterine activity, parity and analgesia, and its causes—inadequate contractions, malposition, cephalopelvic disproportion, maternal exhaustion, or a combination of these—are inferred from the whole clinical picture rather than from an image. Focused intrapartum POCUS should therefore be understood as contributing one component of that picture, typically by clarifying position or presentation, and as a trigger for senior obstetric review rather than as a substitute for it. Used in this way, within the operator’s defined competence and for a question with immediate consequences, focused intrapartum POCUS is a useful adjunct; used as a shortcut around comprehensive intrapartum assessment or around clinical judgment, it is not.

Postpartum and emergency obstetric POCUS remains promising but underevaluated. In postpartum hemorrhage, ultrasound may help identify retained products, intrauterine clot, uterine inversion, bladder distension, balloon tamponade position or free intraperitoneal fluid. However, postpartum hemorrhage is primarily a clinical emergency, and ultrasound must not delay resuscitation, uterotonic therapy, tranexamic acid, manual procedures, surgical escalation or massive transfusion protocols. POCUS should therefore be framed as an adjunct to structured emergency management, not as a gatekeeper for treatment (Table 1).

**Table 1:** Focused OB/GYN POCUS applications: value, limits and escalation triggers

Clinical setting	High-value focused questions	Clinical value	Limits/escalation triggers
Early pregnancy	Intrauterine pregnancy, fetal cardiac activity, free fluid, gross adnexal pathology	Rapid triage of symptomatic patients; may shorten emergency pathways when a viable IUP is seen	Negative or indeterminate scan does not exclude ectopic pregnancy; TVUS and beta-hCG pathway required
Second/third trimester triage	Fetal presentation, fetal number, fetal cardiac activity, placental orientation, gross amniotic fluid	Immediate labor ward and emergency decision support	Not sufficient for anomaly screening, FGR diagnosis, PAS evaluation or Doppler surveillance
Intrapartum care	Fetal head position (occiput anterior/transverse/posterior), presentation and malpresentation, second twin orientation, fetal cardiac activity	Adds objective information before operative vaginal birth or cesarean decision	Does not replace clinical assessment. Quantitative descent parameters, progression measurements and operative-delivery prediction models belong to comprehensive intrapartum sonography and require specific credentialing. POCUS may support, but cannot itself establish, the diagnosis or cause of labor arrest; uncertain findings require senior obstetric review
Postpartum emergency	Retained tissue/clot, balloon position, inversion, free fluid	Can support differential diagnosis during resuscitation	PPH is a clinical emergency; ultrasound must not delay treatment
Gynecologic emergency	Free fluid, IUD position, large adnexal mass, hematometra, gross pelvic infection	Accelerates triage and helps prioritize specialist imaging/surgery	Complex masses, torsion, endometriosis and oncologic risk require formal TVUS/expert assessment

### Clinical applications in gynecology

Gynecologic POCUS has received less systematic study than obstetric POCUS, but has substantial practical relevance. Acute pelvic pain, abnormal uterine bleeding, suspected ectopic pregnancy, adnexal torsion, pelvic inflammatory disease, tubo-ovarian abscess, intrauterine device malposition and postoperative complications are frequent reasons for urgent gynecologic contact. A focused ultrasound examination can rapidly change risk stratification by identifying free fluid, an empty uterus in a patient with positive pregnancy test, a large adnexal mass, marked hematometra, an obvious abscess or an intrauterine device outside the expected fundal position.

The key gynecologic limitation is the central role of transvaginal ultrasound. DEGUM quality standards emphasize that high-resolution transvaginal imaging is the core modality in gynecologic sonography [8]. Most generic handheld devices are optimized for abdominal, vascular, cardiac or musculoskeletal applications rather than for early pregnancy or adnexal detail. For gynecology, endocavitary handheld probes, hygienic reprocessing workflows and appropriate privacy safeguards are therefore not optional; together they determine whether POCUS is clinically useful or merely a low-resolution abdominal triage tool.

Pilot studies in low-resource settings have shown that handheld POCUS can detect selected gynecologic pathology with reasonable sensitivity and specificity, but these data are not sufficient to support unqualified rule-out decisions [21]. More recent work on handheld gynecologic POCUS and novice assessment of adnexal masses illustrates the same pattern: focused imaging and structured algorithms can improve triage, but definitive characterization of complex adnexal lesions remains dependent on standardized expert ultrasound, IOTA/O-RADS-type risk models and clinical context [22, 23].

The intrauterine device is one of the most pragmatic applications of gynecologic POCUS. Identification of a clearly intrauterine device can rapidly guide management when strings are not visible, when pain follows insertion, or when malposition is suspected. Recent evaluations of smartphone-connected handheld ultrasound for IUD position support the feasibility of this approach in selected populations and with trained operators [24]. Nevertheless, subtle embedment, perforation, pregnancy with IUD in situ or complex pain syndromes still require formal evaluation.

A useful clinical framing is therefore to divide gynecologic POCUS into three tiers. Tier 1 comprises simple rule-in tasks with high immediate value, such as identifying obvious free fluid, confirming a clearly intrauterine device, or recognizing an empty uterus with positive pregnancy test as high-risk for ectopic pregnancy. Tier 2 comprises triage tasks that may guide urgency but not definitive diagnosis, such as adnexal mass assessment, suspected torsion or pelvic infection. Tier 3 comprises specialist tasks, such as endometriosis mapping, pelvic floor imaging, ovarian cancer risk stratification, fertility monitoring and complex postoperative assessment; these should not be labeled POCUS unless performed within a formal specialist pathway.

### Technological advancements: handheld devices, AI and tele-ultrasound

The device landscape has changed substantially since early POCUS reviews. The first wave of portable ultrasound focused primarily on size and affordability. The current wave is defined by systems: wireless probes, multi-array devices, tablet-based interfaces, image optimization presets, Doppler functions, cloud archiving, DICOM transfer, PACS/EHR integration, remote supervision and AI modules. This shift has practical importance. A device that produces a reasonable image but cannot save it securely, attach it to the patient record, support quality review or comply with infection control requirements is not an adequate platform for scalable OB/GYN POCUS.

Handheld image quality is now sufficient for many focused obstetric questions. Validation work supports use for fetal presentation, placental localization and amniotic fluid categorization in selected settings [13, 14]. However, image quality varies across devices, probes, body habitus, gestational age, operator experience and clinical target. For OB/GYN, device selection should be indication-driven. A convex probe may suffice for fetal lie and presentation; a high-frequency endocavitary probe is needed for early pregnancy and adnexal imaging; Doppler capability is useful for selected gynecologic and obstetric questions but should not encourage unsupervised fetal Doppler use outside appropriate indications.

AI is the most visible recent advance. The most important POCUS-relevant AI concept in obstetrics is not yet autonomous anomaly diagnosis, but acquisition simplification. Blind-sweep protocols allow lightly trained operators to acquire standardized video sweeps that AI can analyze for gestational age or fetal malpresentation. Gomes and colleagues demonstrated that AI applied to novice-acquired blind sweeps could estimate gestational age noninferiorly to standard fetal biometry and detect malpresentation with high discrimination in low-resource contexts [25]. Slimani and colleagues extended automation by demonstrating end-to-end fetal biometry and amniotic fluid assessment across multiple centers [26]. These developments directly address the most difficult human-resource bottleneck in ultrasound: acquisition and interpretation expertise. Qualitative work with antenatal care stakeholders suggests that the acceptability of AI-enabled obstetric POCUS will depend less on reported accuracy than on clarity about responsibility, training and how results are communicated to pregnant women [27].

Regulatory and implementation developments have accelerated. AI-enabled handheld workflows for gestational age estimation have moved toward real-world deployment and regulatory clearance, with recent reports describing FDA clearance of an AI tool for automated gestational age estimation integrated into a handheld ultrasound platform [28]. Such tools could be transformative in underserved settings, emergency care and regions with low sonographer density. Still, they raise clinical and ethical questions: What population was the model trained on? What gestational age range is supported? How does performance vary by fetal growth restriction, multiple pregnancy, maternal BMI, oligohydramnios or fetal anomalies? Who is responsible when an AI-derived estimate conflicts with clinical dating? What is the escalation pathway for low-confidence results?

Tele-ultrasound is the second major implementation technology. Systematic reviews of tele-ultrasound, handheld and self-operated ultrasound in pregnancy report promising diagnostic agreement for fetal number, presentation, placental location and gestational age in selected settings [29, 30]. Tele-ultrasound can connect midwives, residents, rural clinicians or emergency physicians with remote ultrasound expertise. It can also support asynchronous review of sweeps or clips. Yet it requires stable connectivity, data governance, training, standard acquisition protocols and clarity regarding responsibility. In high-resource European settings, the most realistic tele-ultrasound applications may be hub-and-spoke fetal medicine networks, satellite clinics, labor ward escalation and supervised training rather than fully autonomous patient self-scanning.

### Training, competency assessment and credentialing

POCUS is operator dependent. Its safety depends on the match between the clinical question, device, operator competence and escalation pathway. Training should therefore be indication specific rather than device specific. It is not enough to teach residents how to turn on a handheld probe; they must learn which questions are appropriate for POCUS, which findings are actionable, which findings are indeterminate, and which findings are outside the scope of bedside assessment. The curriculum outline, competency domains and modular credentialing model presented in this and the following section are an expert-informed proposal, developed from the literature reviewed above and from the author’s own teaching and program experience. They have not been formally validated, tested for inter-rater reliability or agreed through a Delphi or other consensus procedure, and they do not represent an endorsed standard of DEGUM, EFSUMB, ISUOG or any national society. They are offered as a starting point for discussion, local adaptation and prospective evaluation, and would require formal consensus development before adoption as a European standard.

A modern OB/GYN POCUS curriculum should include at least four competency domains. The first is image acquisition: probe handling, orientation, optimization, recognition of artifacts and appropriate transabdominal or transvaginal approach. The second is interpretation: recognizing normal findings, actionable abnormal findings and uncertainty. The third is clinical integration: deciding whether the result changes management, requires repetition or mandates escalation. The fourth is professional governance: documentation, consent, data protection, infection control, image storage, quality review and communication of limitations (Table 2).

**Table 2:** The evolution from portable devices to governed POCUS systems

Dimension	Earlier POCUS model	Current/emerging model	Implication for OB/GYN
Device	Portable cart or pocket-sized transabdominal probe	Wireless handheld, multi-array or dedicated endocavitary devices	Device choice must match indication; TVUS remains essential for early pregnancy and gynecology
Image management	Images often not stored or stored locally	Cloud, DICOM, PACS/EHR and enterprise imaging workflows	Documentation and QA become feasible but require governance
AI	Retrospective plane detection and measurement research	Blind-sweep GA estimation, acquisition feedback, automated biometry	AI can reduce acquisition barriers but does not remove clinical responsibility
Telemedicine	Ad hoc image sharing	Live or asynchronous tele-ultrasound with remote expert review	Supports hub-and-spoke care, rural triage and supervised training
Education	Device demonstration and informal supervision	Competency-based modules, simulation, portfolios and audits	Credentialing should be indication specific and longitudinal

Competency assessment is essential. Tolsgaard and colleagues established that ultrasound competence in obstetrics and gynecology can be assessed reliably using structured tools [31]. Consensus recommendations for residency ultrasound curricula similarly emphasize defined learning objectives, simulation and competency-based progression [32]. The 2024 RCOG curriculum guidance further illustrates the international movement from informal exposure to structured ultrasound training in obstetrics and gynecology [33]. In the DACH context, DEGUM provides a mature staged framework that can be used to align POCUS training with existing quality standards rather than create a parallel unregulated pathway [7, 8].

For POCUS, a practical credentialing model should be modular. For example, a labor ward module could include fetal presentation, cardiac activity, placental orientation, amniotic fluid impression and intrapartum head position. An early pregnancy/emergency module would require transvaginal competence and include intrauterine pregnancy, fetal cardiac activity, free fluid and ectopic warning signs. A gynecology triage module would include uterus orientation, endometrium, obvious free fluid, IUD position and major adnexal pathology. Each module should specify minimum supervised scans, image portfolio, direct observation, knowledge assessment, simulation scenarios, refresher intervals and escalation triggers.

### Implementation, quality assurance and medico-legal governance

**Fig. 2:**
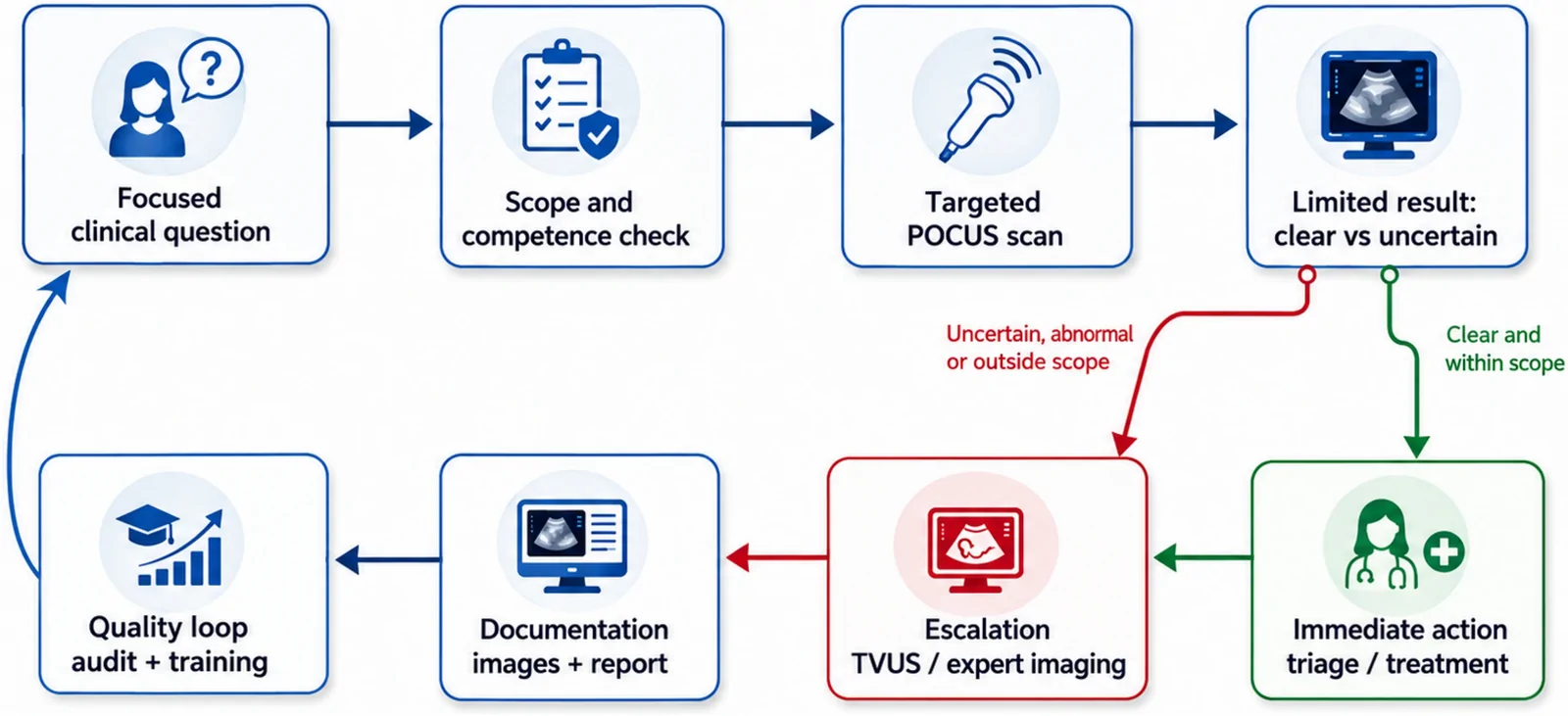
Proposed governance pathway for OB/GYN POCUS. The figure emphasizes that focused scanning should start with a clear question, proceed through scope checking and documentation, and lead either to immediate action or escalation when findings are uncertain, abnormal or outside the operator's defined competence

The next phase of OB/GYN POCUS should be implementation science. Many hospitals can buy handheld devices; far fewer can implement them safely. The governance requirements are predictable: a written scope of practice, device inventory, user authorization, training records, infection control processes, patient consent or information procedures, image storage, report templates, QA review, incident reporting and escalation policies. Without these elements, POCUS risks becoming undocumented medicine (Fig. 2). The individual governance elements listed here are drawn from existing quality traditions and from the POCUS governance literature, but their combination into the OB/GYN-specific framework proposed below is likewise an expert-informed proposal rather than a validated or consensus-tested model.

Documentation is a core quality issue. A POCUS scan that influences clinical management should be recorded in the patient chart with indication, operator, approach, key findings, limitations and subsequent action. Representative images or clips should be stored securely where local policy requires. This is particularly important in obstetrics, where a focused scan might be misinterpreted by patients or clinicians as a complete fetal assessment. Documentation should explicitly state whether the scan was limited and which formal ultrasound follow-up is needed.

Infection control is especially relevant in gynecology because transvaginal probes require validated high-level disinfection or locally approved reprocessing pathways. A handheld endocavitary probe does not simplify this requirement; it may complicate it if the device is used across wards or clinics. Similarly, cloud-based image storage and app-linked devices raise data protection questions under European privacy law. Institutions must know where images are stored, whether identifiers are transmitted, how access is managed and how devices are decommissioned.

Medico-legal risk is reduced when POCUS is framed as a limited examination with documented boundaries. The greatest risk is not that POCUS misses every possible diagnosis; no focused test can do that. The greater risk is that clinicians or patients believe a limited POCUS examination has excluded conditions that were never within its scope. Standard phrases such as "limited POCUS examination for fetal presentation only" or "limited transabdominal POCUS; ectopic pregnancy not excluded" can help align documentation with clinical reality.

Quality assurance should be continuous. Programs should audit image adequacy, documentation completeness, discrepancy rates, escalation appropriateness, adverse events and user retention of competence. Random case review, remote expert feedback, simulation refreshers and complication conferences can make POCUS a learning system. Governance considerations for POCUS in broader health systems emphasize the same principle: safe scale requires integration with enterprise imaging, training and quality infrastructure [34].

### Future directions

The future of OB/GYN POCUS will likely be shaped by five developments. First, endocavitary handheld technology will determine whether gynecologic POCUS can move beyond abdominal triage. True transvaginal capability, robust disinfection, appropriate ergonomics and secure documentation are prerequisites for meaningful gynecologic expansion.

Second, AI will increasingly support acquisition rather than merely interpretation. Real-time sweep quality feedback, standard-plane detection, automated biometry, gestational age estimation and low-confidence alerts are likely to be more clinically useful than black-box anomaly labels. For fetal anomaly detection, AI-enabled POCUS must be approached cautiously: the ethical burden of false reassurance is high, and comprehensive screening requires a different standard than focused triage.

Third, tele-ultrasound will support distributed expertise. The most realistic model is not remote replacement of ultrasound experts, but extension of their supervision: residents in labor wards, rural clinicians, midwives and peripheral clinics can acquire focused clips for immediate or asynchronous review. This model may improve equity if it is embedded in referral pathways. This supervised model should be distinguished from unsupervised self-scanning at home, which professional bodies have advised against because apparent reassurance may be false and no escalation pathway exists [35].

Fourth, education will become more objective. Simulation, standardized assessment tools, image portfolios, AI feedback and structured audit can make ultrasound training more transparent. Given the shortage of ultrasound expertise in many systems, POCUS training should be introduced early in medical education and reinforced during residency, but only with clear separation between focused and comprehensive competencies.

Fifth, outcome research must mature. Many studies demonstrate feasibility or diagnostic agreement; fewer demonstrate improved maternal, fetal or system outcomes. Future trials should evaluate time to diagnosis, unnecessary transfers, length of stay, procedure success, patient satisfaction, cost-effectiveness, safety events, equity of access and downstream imaging burden. Research should also examine when POCUS increases unnecessary follow-up because of false positives or indeterminate findings. The most useful future evidence will be pragmatic, multicenter and pathwaybbased.

## Conclusion

POCUS in obstetrics and gynecology has entered a new phase. The field is no longer defined merely by portable devices. It is defined by focused clinical questions, mobile and increasingly AI-supported acquisition, telemedicine connectivity, structured training and governance. The most clearly useful applications are those that provide immediate, limited and actionable information: early pregnancy triage, fetal viability, fetal presentation, selected placental and amniotic fluid questions, intrapartum head position, IUD localization and emergency gynecologic triage.

At the same time, POCUS must not be used as a shortcut around specialist ultrasound. Early pregnancy complications, fetal growth restriction, placental accreta spectrum, fetal anomalies, complex adnexal masses and oncologic risk stratification require formal imaging pathways. The safest implementation strategy is therefore not to ask whether POCUS can replace existing ultrasound services, but how it can connect patients more rapidly to the right level of ultrasound expertise.

For the European ultrasound community, the next step is to develop OB/GYN POCUS programs that are indication specific, competency based, documented, auditable and aligned with existing DEGUM, ISUOG, EFSUMB and national quality standards. In this model, POCUS is not a device; it is a governed clinical pathway that brings the right ultrasound question to the bedside at the right time. The curriculum, credentialing and governance elements proposed in this review are intended as an expert-informed contribution to that development—a proposal to be debated, adapted and evaluated—and not as a finished or consensus-based European standard.

## Data Availability

No datasets were generated or analyzed during the current study.
